# Supplementary material for: A fault-intrusion-tolerant system and deadline-aware algorithm for scheduling scientific workflow in the cloud
Source: PeerJ Comput Sci. 2021 Nov 2;7:e747. doi: 10.7717/peerj-cs.747 (PMC8576554; doi:10.7717/peerj-cs.747)
Supplement: Supplemental Information 6 [file peerj-cs-07-747-s006.zip › Myproject1.1/docs/org/cloudbus/cloudsim/network/datacenter/NetworkHost.html]

NetworkHost


JavaScript is disabled on your browser.


- Overview
- Package
- Class
- Use
- Tree
- Deprecated
- Index
- Help

- Prev Class
- Next Class

- Frames
- No Frames

- All Classes

- Summary:
- Nested |
- Field |
- Constr |
- Method

- Detail:
- Field |
- Constr |
- Method


org.cloudbus.cloudsim.network.datacenter

## Class NetworkHost

- java.lang.Object
- - org.cloudbus.cloudsim.Host
  - - org.cloudbus.cloudsim.network.datacenter.NetworkHost

- ---

    

  ```
  public class NetworkHost
  extends Host
  ```

  NetworkHost class extends Host to support simulation of networked datacenters. It executes
  actions related to management of packets (send and receive)other than that of virtual machines
  (e.g., creation and destruction). A host has a defined policy for provisioning memory and bw, as
  well as an allocation policy for Pe's to virtual machines.
  Please refer to following publication for more details:
  Saurabh Kumar Garg and Rajkumar Buyya, NetworkCloudSim: Modelling Parallel Applications in Cloud
  Simulations, Proceedings of the 4th IEEE/ACM International Conference on Utility and Cloud
  Computing (UCC 2011, IEEE CS Press, USA), Melbourne, Australia, December 5-7, 2011.

  Since:
  :   CloudSim Toolkit 3.0

- - ### Field Summary

    Fields

    | Modifier and Type | Field and Description |
    | --- | --- |
    | `double` | `bandwidth` |
    | `java.util.List<java.lang.Double>` | `CPUfinTimeCPU` time when last job will finish on CPU1 |
    | `double` | `fintime` |
    | `double` | `memory` |
    | `java.util.List<NetworkPacket>` | `packetrecieved` |
    | `java.util.List<NetworkPacket>` | `packetTosendGlobal` |
    | `java.util.List<NetworkPacket>` | `packetTosendLocal` |
    | `Switch` | `sw` |
  - ### Constructor Summary

    Constructors

    Constructor and Description

    `NetworkHost(int id,
    RamProvisioner ramProvisioner,
    BwProvisioner bwProvisioner,
    long storage,
    java.util.List<? extends Pe> peList,
    VmScheduler vmScheduler)`
  - ### Method Summary

    Methods

    | Modifier and Type | Method and Description |
    | --- | --- |
    | `double` | `getMaxUtilizationAmongVmsPes(Vm vm)` |
    | `double` | `updateVmsProcessing(double currentTime)` Requests updating of processing of cloudlets in the VMs running in this host. |

    - ### Methods inherited from class org.cloudbus.cloudsim.Host

      `addMigratingInVm, allocatePesForVm, deallocatePesForVm, getAllocatedMipsForVm, getAvailableMips, getBw, getBwProvisioner, getDatacenter, getId, getMaxAvailableMips, getNumberOfFreePes, getNumberOfPes, getPeList, getRam, getRamProvisioner, getStorage, getTotalAllocatedMipsForVm, getTotalMips, getVm, getVmList, getVmScheduler, getVmsMigratingIn, isFailed, isSuitableForVm, reallocateMigratingInVms, removeMigratingInVm, setBwProvisioner, setDatacenter, setFailed, setFailed, setId, setPeList, setPeStatus, setRamProvisioner, setStorage, setVmScheduler, vmCreate, vmDeallocate, vmDeallocateAll, vmDestroy, vmDestroyAll`
    - ### Methods inherited from class java.lang.Object

      `clone, equals, finalize, getClass, hashCode, notify, notifyAll, toString, wait, wait, wait`

- - ### Field Detail


    - #### packetTosendLocal

      ```
      public java.util.List<NetworkPacket> packetTosendLocal
      ```


    - #### packetTosendGlobal

      ```
      public java.util.List<NetworkPacket> packetTosendGlobal
      ```


    - #### packetrecieved

      ```
      public java.util.List<NetworkPacket> packetrecieved
      ```


    - #### memory

      ```
      public double memory
      ```


    - #### sw

      ```
      public Switch sw
      ```


    - #### bandwidth

      ```
      public double bandwidth
      ```


    - #### CPUfinTimeCPU

      ```
      public java.util.List<java.lang.Double> CPUfinTimeCPU
      ```

      time when last job will finish on CPU1


    - #### fintime

      ```
      public double fintime
      ```
  - ### Constructor Detail


    - #### NetworkHost

      ```
      public NetworkHost(int id,
                 RamProvisioner ramProvisioner,
                 BwProvisioner bwProvisioner,
                 long storage,
                 java.util.List<? extends Pe> peList,
                 VmScheduler vmScheduler)
      ```
  - ### Method Detail


    - #### updateVmsProcessing

      ```
      public double updateVmsProcessing(double currentTime)
      ```

      Requests updating of processing of cloudlets in the VMs running in this host.

      **Overrides:**
      :   `updateVmsProcessing` in class `Host`

      Parameters:
      :   `currentTime` - the current time

      Returns:
      :   expected time of completion of the next cloudlet in all VMs in this host.
          Double.MAX\_VALUE if there is no future events expected in th is host


    - #### getMaxUtilizationAmongVmsPes

      ```
      public double getMaxUtilizationAmongVmsPes(Vm vm)
      ```


- Overview
- Package
- Class
- Use
- Tree
- Deprecated
- Index
- Help

- Prev Class
- Next Class

- Frames
- No Frames

- All Classes

- Summary:
- Nested |
- Field |
- Constr |
- Method

- Detail:
- Field |
- Constr |
- Method
